# Supplementary material for: Low Replicative Stress Triggers Cell-Type Specific Inheritable Advanced Replication Timing
Source: Int J Mol Sci. 2021 May 7;22(9):4959. doi: 10.3390/ijms22094959 (PMC8125030; doi:10.3390/ijms22094959)
Supplement: Supplementary file 1 [file ijms-22-04959-s001.zip › ijms-1185978-supplementary.pdf]

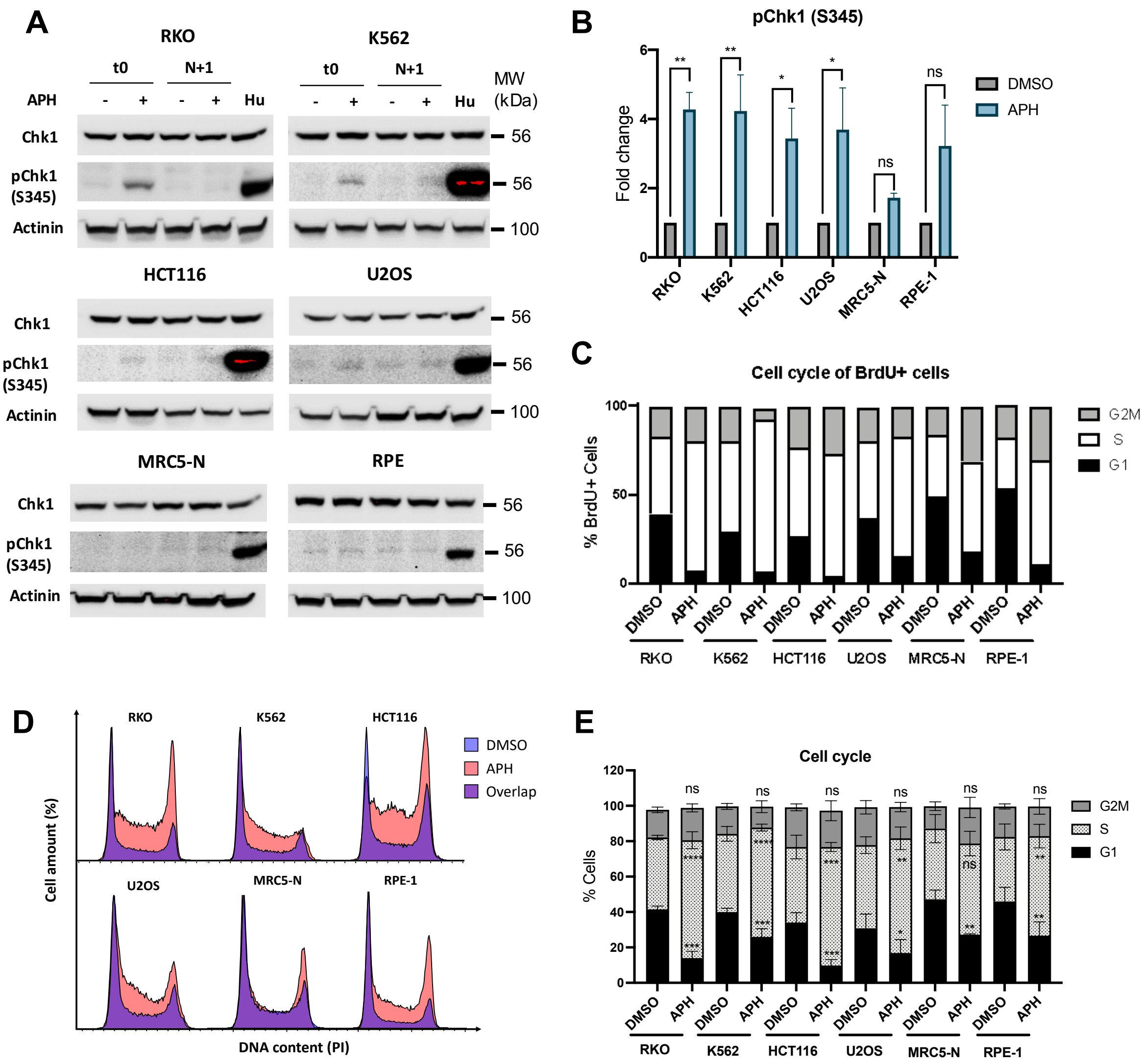

Figure S1. Impact of Aphidicolin on cell cycle and S-phase checkpoint. (**A**) Western blot of pChk1 (Serine 345), Chk1 and Actinin with and without APH for the first generation of cells (t0), daughter cells (N+1) or positive control Hu (2mM, 2h). (**B**) Quantification of p-Chk1 intensity between DMSO and aphidicolin (APH) treatment detected by western blot. Fold change correspond to normalized APH pChk1 intensity (by Chk1 and Actinin intensities) divided by normalized DMSO pChk1 intensity. Statistics (N>4): Two-way ANOVA, Sidak's multiple comparisons test: **$p < 0.01$, *$p < 0.05$, ns when $p > 0.05$. (**C**) Stacked histograms representing the percentage of BrdU positive cells in each cell cycle fraction (G1, S or G2M). Cell cycle analysis was assessed by FACS (DAPI, EdU and BrdU) labeling after BrdU incorporation during the treatment (12-16h) and 15min EdU pulse before cell harvesting and fixation. (**D**) Density plots overlays of DMSO (purple/blue) and APH (pink) density plot showing cell cycle based on PI content by FACS analysis. (**E**) Stacked histograms representing cell cycle repartition (in percentage) between G1, S and G2/M phase. Statistics (N=3): Two-way ANOVA, Classical ****$p < 0.0001$, ***$p < 0.005$, **$p < 0.01$, *$p < 0.05$, ns when $p > 0.05$.

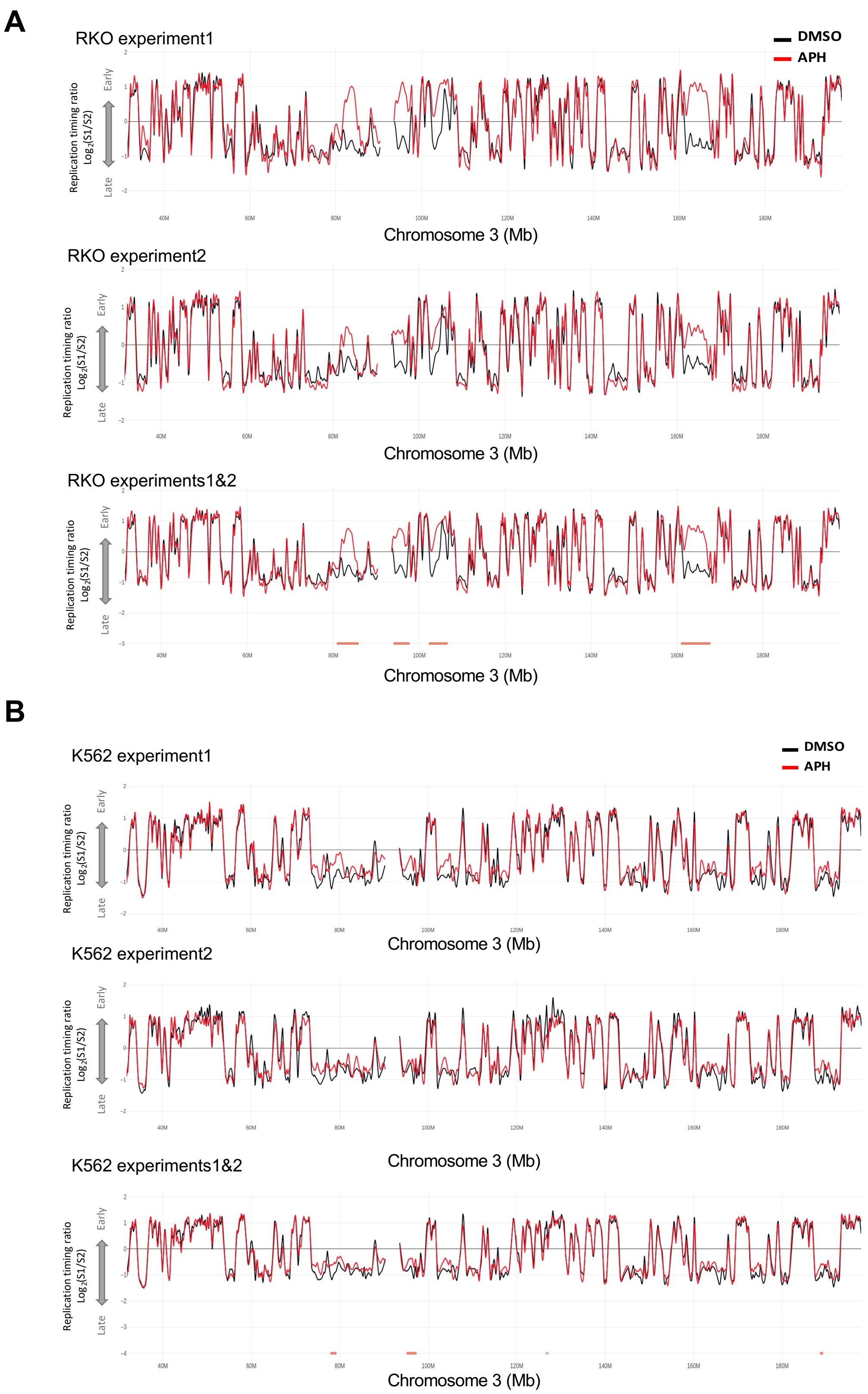

Figure S2. Low replication stress induces cell-type replication timing changes. Screenshot of Loess-smooth RKO (**A**) and K562 (**B**) RT profiles for the same region of Chromosome 3 in two independent biological replicates and combined replicates analysed with the START-R software allowing to highlight significant ADV aRTIL (orange tracks) and DEL aRTIL (grey tracks). The dark lines correspond to replication timing of control (DMSO) replication timing and the red lines are replication timing of APH treated cells.

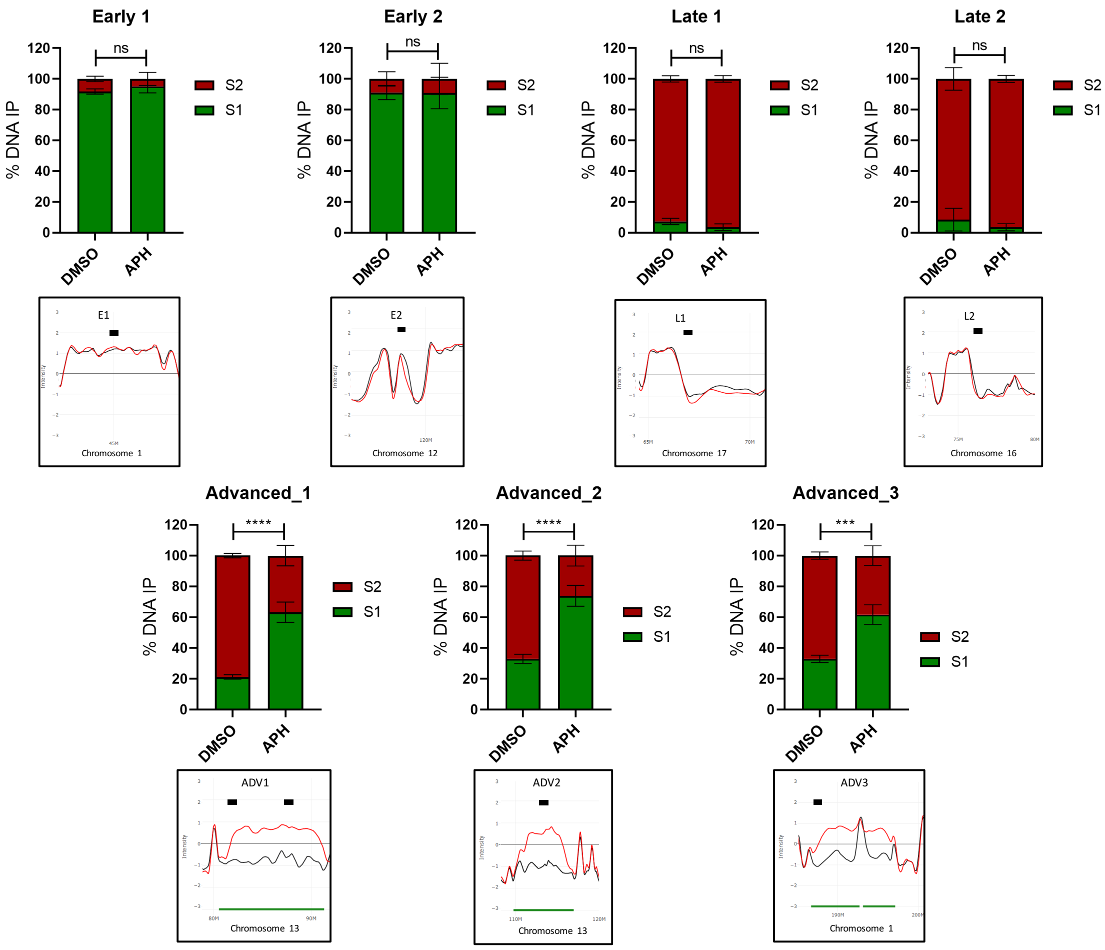

Figure S3. Validation of ADV aRTIL by BrdU-ChIP-qPCR. The experiment was performed in RKO cells, after cell cycle sorting between early (S1) and late (S2) S phase. Stacked histograms represent the % of DNA IP for a given genomic domain in the S1 or S2 fraction. Under each histogram, there is a screenshot of Loess-smooth RT profiles in the given region for DMSO (black) and APH (red) conditions, the used oligo is underlined in black. The two first histograms recapitulates results for the two early control domains (E1 and E2), the third and fourth histograms recapitulates the results for the two late control domains (L1, L2) and the last ones for the three advanced regions in the aphidicolin condition (ADV aRTIL) (D1, D2, D3). Statistics (three biological replicates, N=3): Two-way ANOVA, Sidak's multiple comparison test **** $p < 0.0001$, ns when $p > 0.05$.

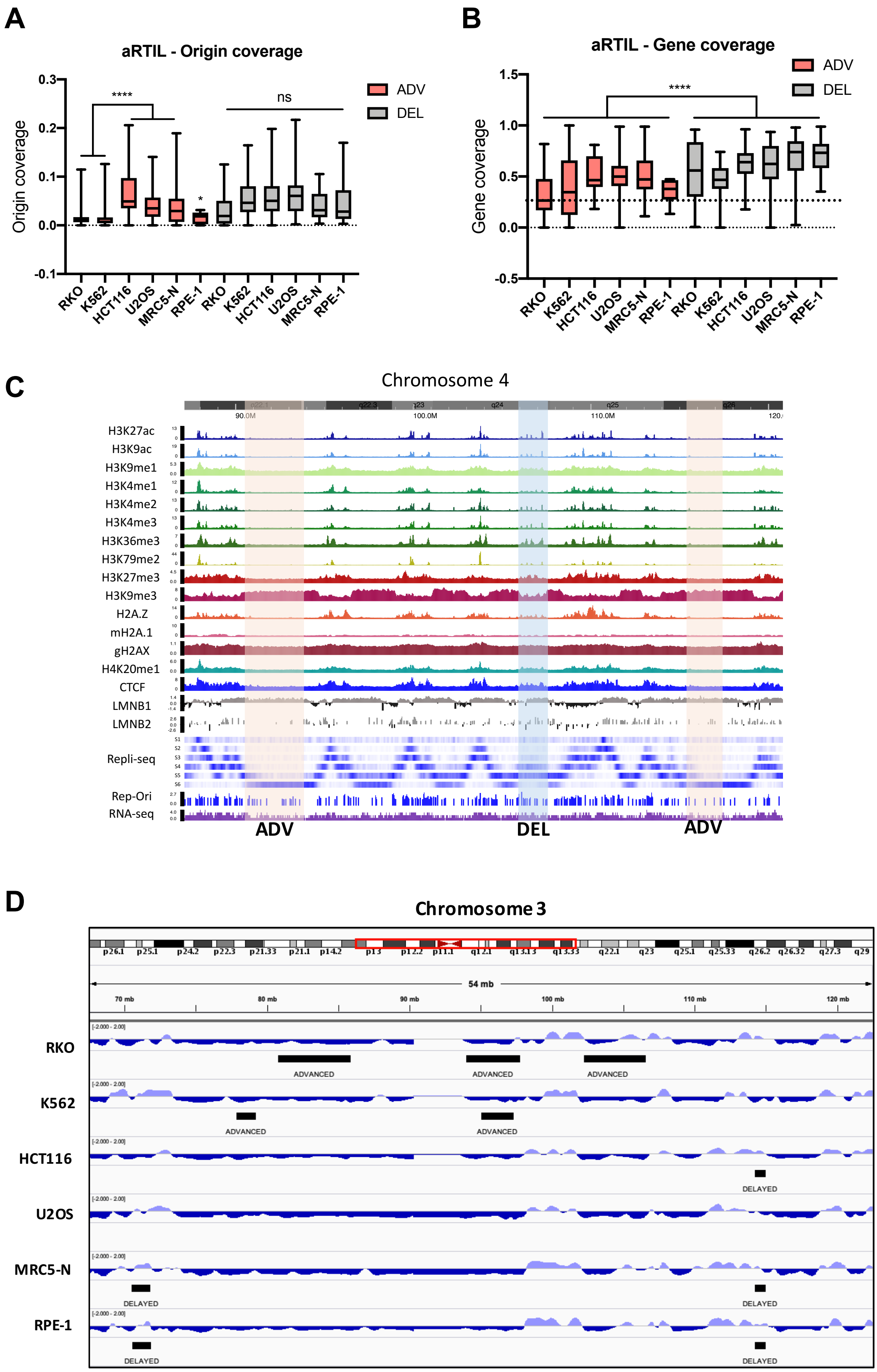

Figure S4. Genomic and epigenomic characteristics of aRTIL. Box and whiskers (min to max) representing (**A**) constitutive origins coverage and (**B**) gene coverage within ADV and DEL aRTIL in the 6 cell lines. Statistics: Kruskal-Wallis test with Dunn's multiple comparison: ****p < 0.0001, **p < 0.01, *p < 0.05, ns p > 0.05. (**C**) Screenshot of WashUEpigenome Browser for chromosome 4 in K562 with an example of one DEL aRTIL (highlighted in blue) and two examples of ADV aRTIL (highlighted in orange). (**D**) IGV snapshot of normal replication timing (bedgraph files, light purple = early, dark purple = late), ADV and DEL aRTIL (black tracks) in the 6 cell lines (Chromosome 3).

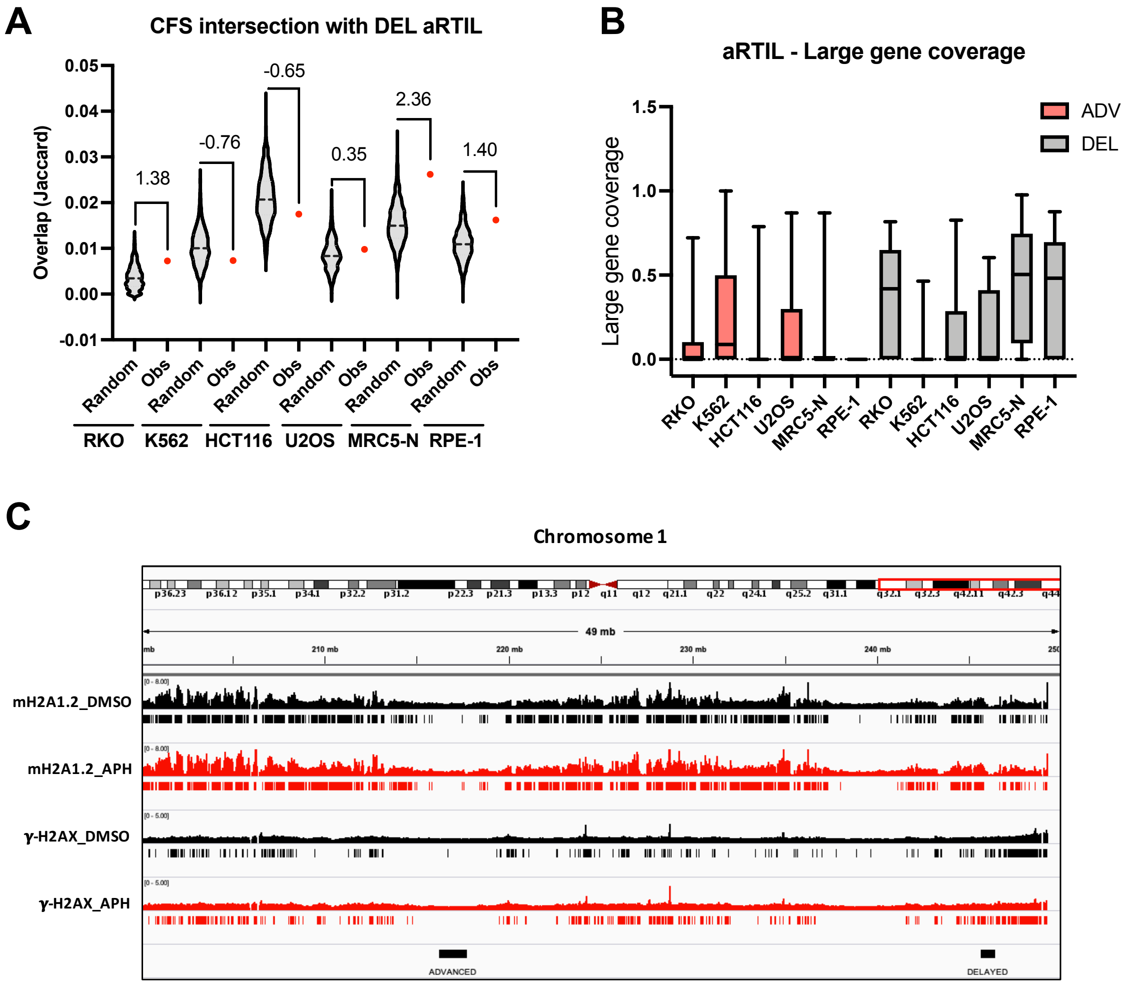

Figure S5. ADV aRTIL are closely related to CFS but are not targeted by DNA damage signalling. (**A**) Jaccard index measuring overlaps of CFS with observed DEL aRTIL (red), and randomly shuffled regions of equal size (grey). Values of z-score between random and observed Jaccard are indicated as statistics. (**B**) Box and whiskers (min to max) representing large gene coverage within ADV and DEL aRTIL in the 6 cell lines. (**C**) IGV snapshot of ChIP-seq data (bigwig and bed files) for γ-H2AX and mH2A1.2 histone modifications coverage for DMSO (black) and APH (red) treated conditions within Chromosome 1 regions that includes ADV and DEL aRTIL.

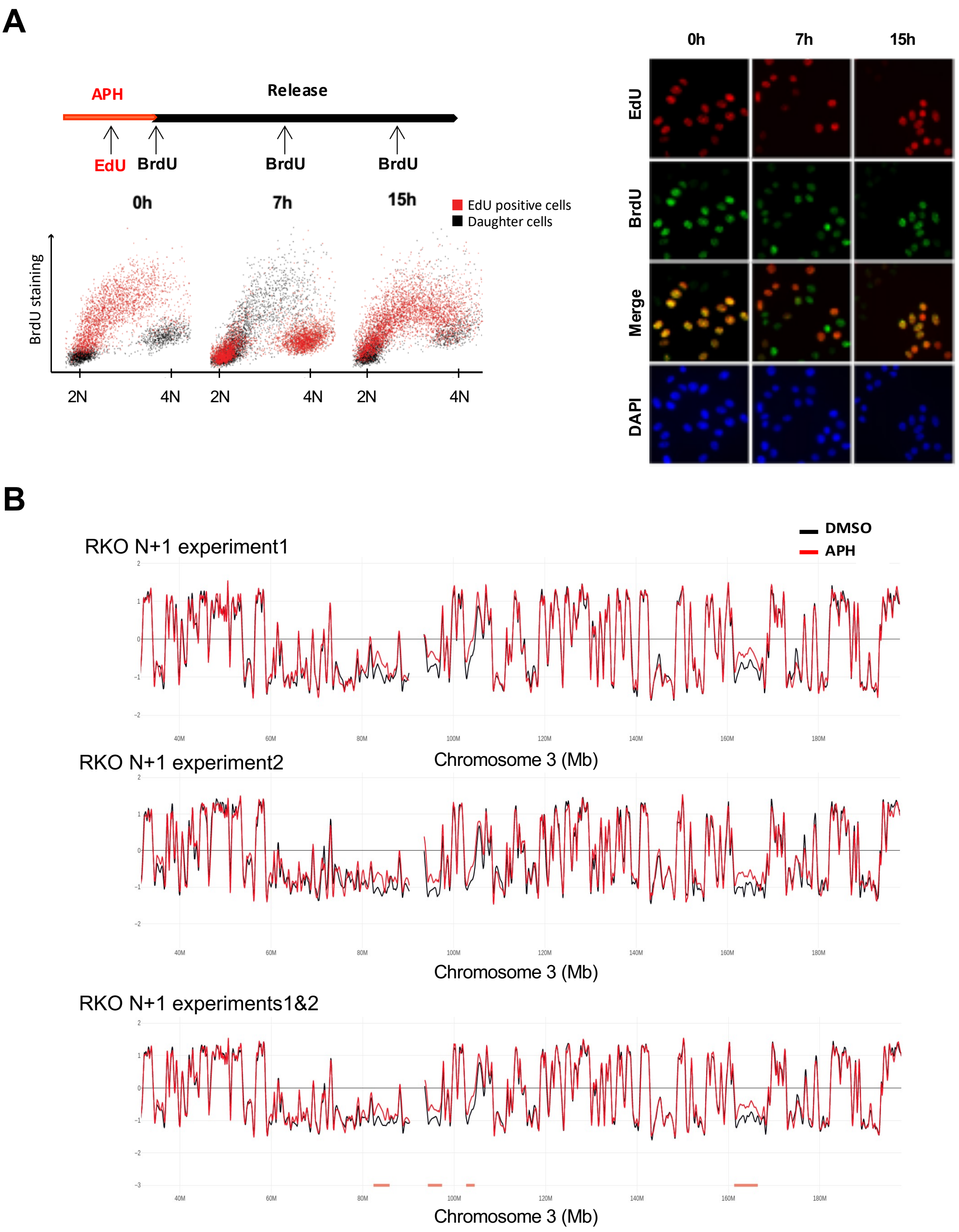

Figure S6. Experimental set-up and replication timing visualization in RKO daughter cells. (**A**) Cells were pulsed with EdU at the end of the APH treatment, in order to label stressed mother cells and released in absence of APH for different durations. At the end of the release, BrdU was added to the media, in order to label S phase cells at the time of collection. DAPI, BrdU and EdU intensities were measured by microscopy at different time after release and EdU positive single cells (in red) were plotted on the cell cycle graph (DAPI vs BrdU) thanks to QiBC approach (see method). (**B**) Screenshot of Loess-smooth RT profiles in RKO daughter cells (N+1) for the same region of Chromosome 3 in two independent biological replicates and combined replicates analysed with the START-R software allowing to highlight significant ADV aRTIL (orange tracks). The dark lines correspond to replication timing of control (DMSO) replication timing and the red lines are replication timing of APH treated cells.

A

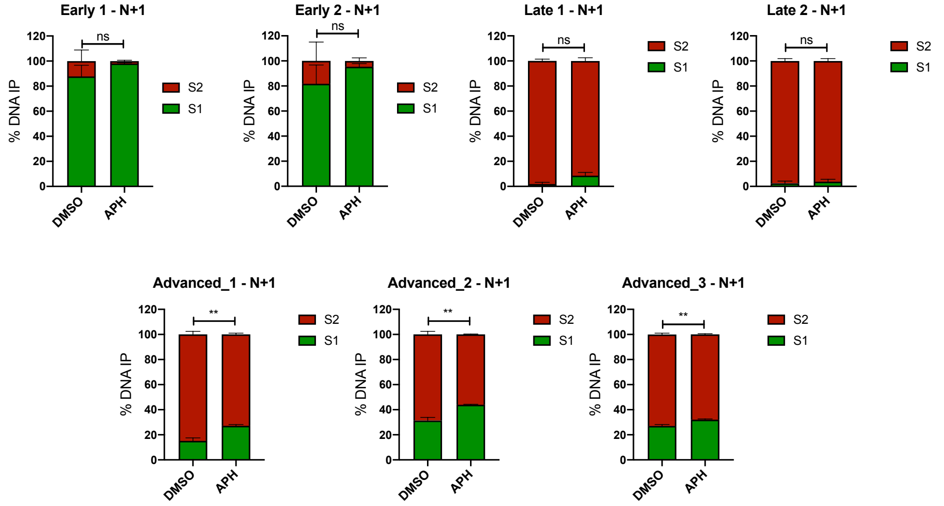

B

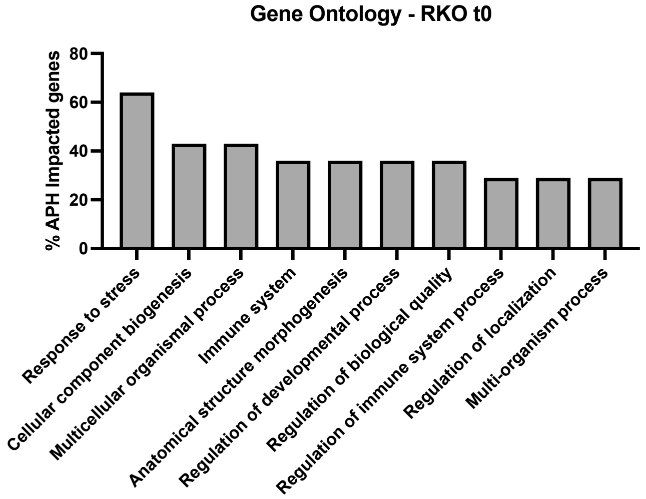

Figure S7. ADV aRTIL can be transmitted to daughter cells. (**A**) BrdU-ChIP-qPCR in RKO daughter cells (N+1) after release from aphidicolin and cell cycle sorting between early (S1) and late (S2) S phase. Stacked histograms represent the % of DNA IP for a given genomic domain in the S1 or S2 fraction. The two first histograms recapitulates results for the two early control domains (E1 and E2), the third and fourth histograms recapitulates the results for the two late control domains (L1, L2) and the last ones for the three advanced regions in the aphidicolin condition (ADV aRTIL) (D1, D2, D3). Statistics (three biological replicates, N=3): Two-way ANOVA, Sidak's multiple comparison test ****$p < 0.0001$, ns when $p > 0.05$. (**B**) Histogram of Gene Ontology classification for significantly impacted genes under aphidicolin treatment in RKO mother cells (t0). The y axis represent the percentage of impacted genes. The top 10 of the most significant biological process (based on FDR value, FDR < 0.05) is represented in x axis.

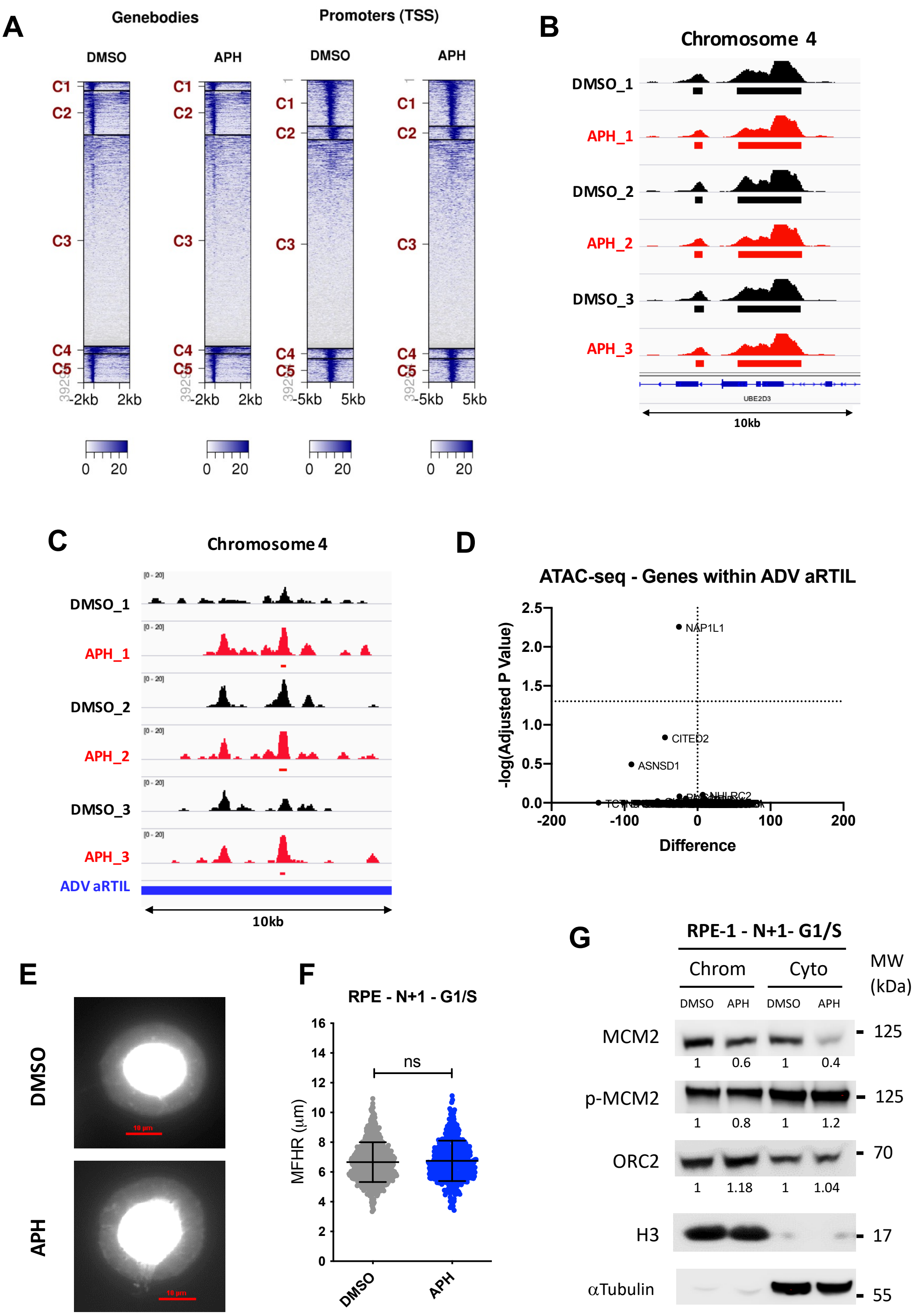

Figure S8. Aphidicolin modulates chromatin accessibility in RKO cells. (**A**) Heatmaps of ATAC-seq peak average values within gene bodies (left) and promoters (right). Values in z-axis (higher in blue, lowest in white), regions in y-axis. Data are clustered (C1- C5) for quality control. IGV snapshot of ATAC-seq triplicates (N = 3) bigwig files within (**B**) a random promoter (UBE3D3 gene) and (**C**) a specific ADV aRTIL in chromosome 4. (**D**) Volcano plot for differential ATAC-seq average peak values within ADV aRTIL genes. (**E**) Visualization (red scale = 10 µm) and (**F**) Quantification of DNA Halo size (MFHR in µm) in RPE-1 G1/S synchronized daughter cells released from DMSO or aphidicolin treatment. Error bars: SD. Statistics (N=3): Unpaired t-test, Welch's correction ns when $p < 0.05$. (**G**) Western blot on chromatin (Chrom) and cytoplasmic (Cyto) protein fractions to quantify the amount of MCM2, p-MCM2 and ORC2 in the G1/S cells (synchronized with L-mimosine). The mean fold change between DMSO and APH conditions is reported on the figure. H3 and tubulin values were used to normalise protein signal in chromatin and cytoplasmic fraction, respectively.

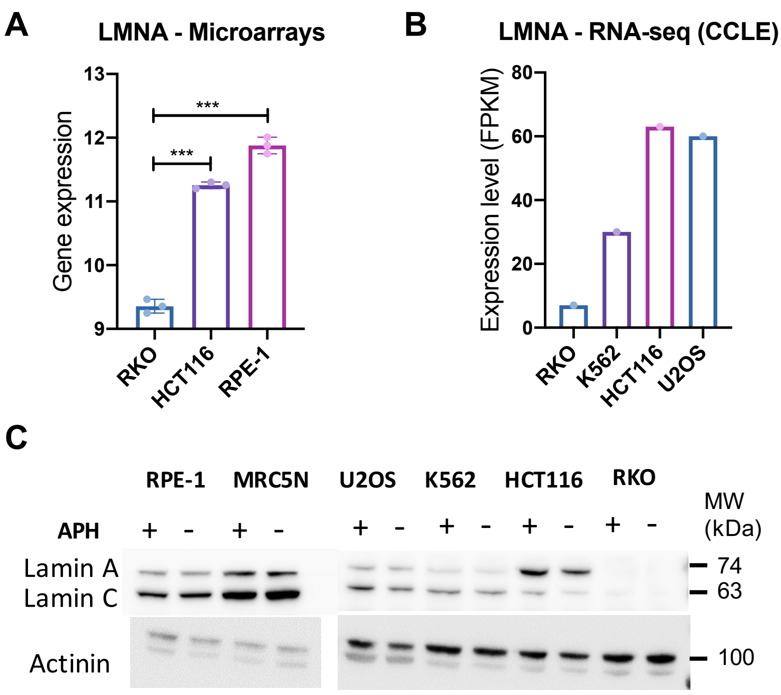

Figure S9. Gene expression (LMNA) and protein level of LaminA/C in cancer and non-cancer cells. (**A**) Gene expression of LMNA gene from microarrays in RKO, HCT116 and RPE-1. Statistics: Two-way ANOVA, Tukey's multiple comparisons test ****$p < 0.0001$. (**B**) Expression level (FPKM) of LMNA gene expression from RNA-seq in RKO, K562, HCT116 and U2OS (values from the CCLE data set). (**C**) Western Blot for LaminA/C and actinin (for loading control) protein in the six cell lines aphidicolin treated (APH+) or not (APH-).

Supplemental tables

Table S1. Cellular and molecular characteristics of the 6 human cell lines used for the study. Cellular background regarding differentiation state, morphology and finally some molecular characteristics like p53 status or telomere maintenance mechanism are listed in this table. MMR: Mismatch repair, CIN: chromosome instability.

Cell line	RKO	HCT116	U2OS	K562	MRC5-N	RPE-1
**Gender**	Female	Male	Female	Female	Male	Female
**Cancer type**	Carcinoma	Carcinoma	Osteosarcoma	Leukemia	Non-cancerous	Non-cancerous
**Tissue**	Colon	Colon	Bone	Bone marrow	Lung	Retina
**Cell type**	Epithelial	Epithelial	Epithelial	Hematopoietic	Fibroblast	Epithelial
**State of differentiation**	Poorly differentiated	Poorly Unable to differentiate	Moderately differentiated	Undifferentiated progenitor	Embryonic	Terminally differentiated
**Molecular characteristics**	p53+ (WT)<br>MMR-<br>Telomerase<br>CIN-	p53+ (WT)<br>MMR-<br>Telomerase<br>CIN-	p53+ (WT)<br>MMR+<br>ALT<br>CIN+	p53-<br>MMR+<br>Telomerase<br>CIN+	p53+ (WT)<br>MMR+<br>Normal<br>CIN-	p53+ (WT)<br>MMR+<br>hTERT<br>CIN-

Table S2. Treatment and release duration for the six cell lines.

	RKO	K562	HCT116	U2OS	MRC5-N	RPE-1
**Time of treatment (t0)**	16h	12h	15h	15h	15h	12h
**Time of release (N+1)**	13h	18h	14h	15h	12h	13h

Table S3. Names, genomic sequences and location (hg19) of BrdU-ChiP-qPCR probes.

Name	Forward sequence	Reverse sequence	Chr	Start	End
**L1**	CCCCATCCCCAGTTCTTTCC	TGGTGGGACTTGTGCTGTTT	chr17	69565311	69565341
**L1bis**	TGGCACGTTCTTGTCACACT	AAGGTCCTCAGCCATTCAGC	chr17	69617443	69617473
**L2**	TCACTGCCAGTTCGACACAG	ACCCAGTCCCACATCACTCT	chr16	76802979	76803009
**L2bis**	TGTGTCCATGCTGTGCCTAG	TGATGGAAGCAGCTACGTGG	chr16	76697788	76697818
**E1**	TGACTTCCGCTTCGAACCTC	GATGCTTGCACTCCCTCTGT	chr1	45285669	45285699
**E2**	TCCATCCTCAGGTCCTCGAG	AATGGCACGGTTCTCAGGAG	chr12	121471353	121471383
**ADV1**	GTTTCCTGGATGTTGCGCAG	CCAACAGAGACCCAGCAGAG	chr13	89558052	89558082
**ADV1bis**	CAGCTTCACAGACCTCTCCG	GCTTGCATCACTGGAGTCGA	chr13	82986981	82987011
**ADV2**	GCCAAGCTCGCACAATGTTT	TCTTTCCACTTGACACTGGG	chr8	114399270	114393300
**ADV2bis**	GTTGCCCCATCACGAAAACC	GCATGACCCTGTATCTGCCC	chr8	114309051	114309081
**ADV3**	ATGGATTAGGCCCCGGTACT	CTCACTGCAATCCTGACGGT	chr1	188813945	188813975
**ADV3bis**	GAAGCTTCAGAGGCCGACAT	GGAGACCATCAATGCCCGTT	chr1	188768169	188768199
**Mito**	CCTAGGAATCACCTCCCATTCC	GTGTTTAAGGGGTTGGCTAGGG	/	/	/

Table S4. Reference of public ChIP-seq data used in this study.

Histone mark	K562	HCT116
**H3K4me1**	ENCFF320NKZ	ENCSR161MXP
**H3K27ac**	ENCFF770UZZ	ENCSR661KMA
**H3K36me3**	ENCFF676RW	ENCSR091QXP
**H3K27me3**	ENCFF369ZKL	ENCSR810BDB
**H3K9ac**	ENCFF842GQO	ENCSR093SHE
**H3K4me3**	ENCFF729NQN	ENCSR333OPW
**H3K9me3**	GSM607494	ENCSR179BUC
**H3K79me2**	ENCFF520WBR	ENCSR49ACCN
**H3K4me2**	ENCFF298QOP	ENCSR794ULT
**DNase-seq**	ENCFF433TIR	ENCSR000ENM
**LaminB1 (Dam-ID)**	4DNFIHGCLSYW	4DNFI6JL1IPW
**γ-H2AX_NT**	GSM2808044	/
**γ-H2AX_APH**	GSM2808042	/
**mH2A1.2_NT**	GSM2808015	/
**mH2A1.2_APH**	GSM2808019	/

Table S5. Impacted genes in RKO daughter cells and their link to cancer. List of the 17 APH UP genes and 3 APH DOWN genes (marked by a star*) in RKO daughter cells (N+1) and their link with cancer (NR=Not reported).

Gene name	Description	Cancer related	Reference
**APOC1**	Encodes a member of the apolipoprotein C1 family. This gene is expressed primarily in the liver, and it is activated when monocytes differentiate into macrophages.	Yes	The Human Protein Atlas (Ren et al., 2019)
**CRABP2**	Encodes a member of the retinoic acid (RA, a form of vitamin A) binding protein family and lipocalin/cytosolic fatty-acid binding protein family. The protein is a cytosol-to-nuclear shuttling protein, which facilitates RA binding to its cognate receptor complex and transfer to the nucleus.	Yes	The Human Protein Atlas (Feng et al., 2019) (Wu et al., 2019)
**IFI6**	First identified as one of the many genes induced by interferon. The encoded protein may play a critical role in the regulation of apoptosis.	Yes	The Human Protein Atlas (Qi et al., 2015) (Cheriyath et al., 2018)
**MGLL**	Converts monoacylglycerides to free fatty acids and glycerol. Regulates cannabinoid receptor 2-dependent macrophage activation and promote cancer cell migration, invasion and tumour growth.	Yes	The Human Protein Atlas (Xiang et al., 2018)
**KRTAP2-1**	Hair keratin-associated protein (KRTAP), essential for the formation of a rigid and resistant hair shaft through their extensive disulfide bond cross-linking with abundant cysteine residues of hair keratins.	NR	The Human Protein Atlas
**KRTAP2-2**	Hair keratin-associated protein (KRTAP), essential for the formation of a rigid and resistant hair shaft through their extensive disulfide bond cross-linking with abundant cysteine residues of hair keratins.	NR	The Human Protein Atlas
**PDGFRL**	Platelet derived growth factor receptor like. This gene encodes a protein with significant sequence similarity to the ligand binding domain of platelet-derived growth factor receptor beta.	Yes	The Human Protein Atlas (Jiang et al., 2018) (Manzat Saplacan et al., 2017)
**IER3**	Immediate early response 3. This gene may play a role in the ERK signalling pathway by inhibiting the dephosphorylation of ERK. Acts also as an ERK downstream effector mediating survival.	Yes	The Human Protein Atlas (Ye et al., 2018) (Arlt and Schäfer., 2011)

**ARHGAP29**	Rho GTPase activating protein 29. GTPase activator for the Rho-type GTPases by converting them to an inactive GDP-bound state.	Yes	The Human Protein Atlas (Qiao et al., 2017)
**PDP1**	Pyruvate dehydrogenase phosphatase catalytic subunit 1. Catalyses the dephosphorylation and concomitant reactivation of the alpha subunit of the E1 component of the pyruvate dehydrogenase complex.	Yes	The Human Protein Atlas (Kim et al., 2016) (Duan, 2006)
**TMEM136**	Transmembrane protein 136. This gene's in vivo function is yet unknown.	Yes	The Human Protein Atlas
**PSMB9**	Proteasome subunit beta 9. Expression of this gene is induced by gamma interferon and this gene product replaces catalytic subunit 1 (proteasome beta 6 subunit) in the immunoproteasome.	Yes	The Human Protein Atlas (Rouette et al., 2016) (Whitehead et al., 2005)
**TMCO2**	Transmembrane and coiled-coil domains 2. This gene's in vivo function is yet unknown.	NR	The Human Protein Atlas
**NPC2**	NPC intracellular cholesterol transporter 2. The encoded protein may function in regulating the transport of cholesterol through the late endosomal/lysosomal system.	Yes	The Human Protein Atlas (Liao et al., 2013)
**MIR4709**	Overexpression facilitates cancer proliferation and invasion and is an unfavourable prognosis marker in colon cancer	Yes	(Yu et al., 2019)
**IGFBP6**	Insulin like growth factor binding protein 6. Activates the MAPK signalling pathway and induces cell migration.	Yes	The Human Protein Atlas (Nikulin et al., 2018)
**EGFR***	Epidermal growth factor receptor. This protein is a receptor for members of the epidermal growth factor family. EGFR is a cell surface protein that binds to epidermal growth factor.	Yes	The Human Protein Atlas (Spano et al., 2005)
**SLC7A11***	Solute carrier family 7 member 11. This gene encodes a member of a heteromeric, sodium-independent, anionic amino acid transport system that is highly specific for cysteine and glutamate.	Yes	The Human Protein Atlas (Ji et al., 2018)
**KIF14***	Kinesin family member 14. This gene encodes a member of the kinesin-3 superfamily of microtubule motor proteins. These proteins are involved in numerous processes including vesicle transport, chromosome segregation, mitotic spindle formation, and cytokinesis.	Yes	The Human Protein Atlas (Li et al., 2017) (Zhang et al., 2017)

R code: replication timing data hierarchical clustering

```
## Clustering approach_ALL_RT
library(pvclust)
RT_clus <- read.delim2("~/Desktop/R Studio/RT_clus.txt")
RKO_DMSO_clus <- NULL; RKO_APH_clus <- NULL; K562_DMSO_clus <- NULL; K562_APH_clus <- NULL;
HCT116_DMSO_clus <- NULL; HCT116_APH_clus <- NULL; U2OS_DMSO_clus <- NULL; U2OS_APH_clus <-
NULL; MRC5N_DMSO_clus <- NULL; MRC5N_APH_clus <- NULL; RPE_DMSO_clus <- NULL; RPE_APH_clus <-
NULL # initialize variables to store averged data
nWin <- 35  # 5.8 kb median probe spacing * 35 = 203 kb
mLength <- nrow(RT_clus)/nWin # calculate number of windows
for (x in 1:mLength) { # for each potential windows
  z1 <- x * nWin  # determine probe number at window start
  z2 <- (x+1) * nWin  # determine probe number at window end
  RKO_DMSO_clus[x] <- RT_clus$RKO_DMSO[z1:z2]
  RKO_APH_clus[x] <- RT_clus$RKO_APH[z1:z2]
  K562_DMSO_clus[x] <- RT_clus$K562_DMSO[z1:z2]
  K562_APH_clus[x] <- RT_clus$K562_APH[z1:z2]
  HCT116_DMSO_clus[x] <- RT_clus$HCT116_DMSO[z1:z2]
  HCT116_APH_clus[x] <- RT_clus$HCT116_APH[z1:z2]
  U2OS_DMSO_clus[x] <- RT_clus$U2OS_DMSO[z1:z2]
  U2OS_APH_clus[x] <- RT_clus$U2OS_APH[z1:z2]
  MRC5N_DMSO_clus[x] <- RT_clus$MRC5.N_DMSO[z1:z2]
  MRC5N_APH_clus[x] <- RT_clus$MRC5.N_APH[z1:z2]
  RPE_DMSO_clus[x] <- RT_clus$RPE.1_DMSO[z1:z2]
  RPE_APH_clus[x] <- RT_clus$RPE.1_APH[z1:z2]
  cat("Current window:", x ,"/", mLength, "\n")  # write the current window to the console
}   # end for loop

RTWind <- data.frame(RKO_DMSO_clus, RKO_APH_clus, K562_DMSO_clus, K562_APH_clus,
HCT116_DMSO_clus, HCT116_APH_clus, U2OS_DMSO_clus, U2OS_APH_clus, MRC5N_DMSO_clus,
MRC5N_APH_clus,  RPE_DMSO_clus, RPE_APH_clus) # write the result to a new dataframe
cluster.bootstrap <- pvclust(RTWind, method.hclust= "average", method.dist = "correlation",
use.cor="pairwise.complete.obs", nboot = 1000, parallel=FALSE, r=seq(.5,1.4,by=.1),store=FALSE,
weight=FALSE, iseed=NULL, quiet=FALSE)

# plot the cluster dendogram
plot(cluster.bootstrap) # overall dendogram
pvrect(cluster.bootstrap) # outline data sets that cluster at a significant level

# download csv file
dir.create("Rescaled_RT_files")
write_csv(RTWind, "RT_rescaled.csv")


# DMSO
RT_clus <- read.delim2("~/Desktop/R Studio/RT_clus.txt")
RKO_DMSO_clus <- NULL; K562_DMSO_clus <- NULL; HCT116_DMSO_clus <- NULL; U2OS_DMSO_clus <-
NULL; MRC5N_DMSO_clus <- NULL; RPE_DMSO_clus <- NULL # initialize variables to store averged
data
nWin <- 35  # 5.8 kb median probe spacing * 35 = 203 kb
mLength <- nrow(RT_clus)/nWin # calculate number of windows
for (x in 1:mLength) { # for each potential windows
  z1 <- x * nWin  # determine probe number at window start
  z2 <- (x+1) * nWin  # determine probe number at window end
  RKO_DMSO_clus[x] <- RT_clus$RKO_DMSO[z1:z2]
  K562_DMSO_clus[x] <- RT_clus$K562_DMSO[z1:z2]
  HCT116_DMSO_clus[x] <- RT_clus$HCT116_DMSO[z1:z2]
  U2OS_DMSO_clus[x] <- RT_clus$U2OS_DMSO[z1:z2]
  MRC5N_DMSO_clus[x] <- RT_clus$MRC5.N_DMSO[z1:z2]
  RPE_DMSO_clus[x] <- RT_clus$RPE.1_DMSO[z1:z2]
  cat("Current window:", x ,"/", mLength, "\n")  # write the current window to the console
}   # end for loop

RTWind_DMSO <- data.frame(RKO_DMSO_clus, K562_DMSO_clus, HCT116_DMSO_clus, U2OS_DMSO_clus,
MRC5N_DMSO_clus,  RPE_DMSO_clus) # write the result to a new dataframe
cluster.bootstrap_DMSO <- pvclust(RTWind_DMSO, method.hclust= "average", method.dist =
"correlation", use.cor="pairwise.complete.obs", nboot = 1000, parallel=FALSE,
r=seq(.5,1.4,by=.1),store=FALSE, weight=FALSE, iseed=NULL, quiet=FALSE)

# plot the cluster dendogram
```

```
plot(cluster.bootstrap_DMSO) # overall dendogram
pvrect(cluster.bootstrap_DMSO) # outline data sets that cluster at a significant level

# APH
RT_clus <- read.delim2("~/Desktop/R Studio/RT_clus.txt")
RKO_DMSO_clus <- NULL; K562_DMSO_clus <- NULL; HCT116_DMSO_clus <- NULL; U2OS_DMSO_clus <-
NULL; MRC5N_DMSO_clus <- NULL; RPE_DMSO_clus <- NULL # initialize variables to store averged
data
nWin <- 35  # 5.8 kb median probe spacing * 35 = 203 kb
mLength <- nrow(RT_clus)/nWin # calculate number of windows
for (x in 1:mLength) { # for each potential windows
  z1 <- x * nWin  # determine probe number at window start
  z2 <- (x+1) * nWin  # determine probe number at window end
  RKO_DMSO_clus[x] <- RT_clus$RKO_DMSO[z1:z2]
  K562_DMSO_clus[x] <- RT_clus$K562_DMSO[z1:z2]
  HCT116_DMSO_clus[x] <- RT_clus$HCT116_DMSO[z1:z2]
  U2OS_DMSO_clus[x] <- RT_clus$U2OS_DMSO[z1:z2]
  MRC5N_DMSO_clus[x] <- RT_clus$MRC5.N_DMSO[z1:z2]
  RPE_DMSO_clus[x] <- RT_clus$RPE.1_DMSO[z1:z2]
  cat("Current window:", x ,"/", mLength, "\n")  # write the current window to the console
}   # end for loop

RTWind_APH <- data.frame(RKO_APH_clus, K562_APH_clus, HCT116_APH_clus, U2OS_APH_clus,
MRC5N_APH_clus,  RPE_APH_clus) # write the result to a new dataframe
cluster.bootstrap_APH <- pvclust(RTWind_APH, method.hclust= "average", method.dist =
"correlation", use.cor="pairwise.complete.obs", nboot = 1000, parallel=FALSE,
r=seq(.5,1.4,by=.1),store=FALSE, weight=FALSE, iseed=NULL, quiet=FALSE)

# plot the cluster dendogram
plot(cluster.bootstrap_APH) # overall dendogram
pvrect(cluster.bootstrap_APH) # outline data sets that cluster at a significant level
```

Supplemental materials and methods

Fluorescence Activated Cell Sorting (FACS)

Cells were pulse-labelled with 10μM BrdU and/or EdU for indicated times then collected by trypsinization and fixed in 70% ice-cold ethanol overnight at −20 °C. For EdU and BrdU detection, we followed the protocol described in Bradford and Clarke 2011 (Bradford and Clarke, 2011). Finally, after washing in PBS–BSA 0.5%, then in PBS, cells were resuspended in PBS with propidium iodide (25 μg/mL, Invitrogen, p3566) and RNase A (100 μg/mL, Thermo Fisher Scientific, ENO531) or with DAPI (1/1000, Sigma-Aldrich, D9542). After 20 min of incubation at room temperature, cell cycle analysis was carried out by flow cytometry with a MACSQuant 10 or VYB cytometer (Miltenyi Biotec) and analysed with FlowLogic software.

QiBC EdU-BrdU

RKO cells were grown on glass coverslips and treated with APH 0.2μM. At the end of the treatment, cells were incubated for 20 min with EdU 10μM (from Click-it EdU, Molecular probes) and released without APH. At the indicated time points, cells were incubated with BrdU 10μM (Sigma Aldrich) for 20 min. After a wash in PBS, cells were fixed in PBS 4% PFA for 15 min and incubated with 0.5% Triton X-100 in PBS for 5 min. Cells were blocked 30 min with PBS 5% BSA and Click-it EdU reaction was performed according manufacturer instructions (Thermo Fisher, Invitrogen). Then BrdU detection was performed using Monoclonal anti-Bromo-deoxyuridine antibody (clone BU-1) (Amersham, RPN202) according manufacturer instruction, followed by incubation with Alexa Fluor 555 goat anti-mouse (1:1000, Molecular Probes). Finally, cells were incubated for 10 min in DAPI 4μM and coverslips were mounted on microscopy-slides using ProLong Diamond (Molecular Probes). Images acquisition of multiple random fields were carried out on a wide field Nikon EclipseNi microscope 60X objective with oil immersion camera Ds-Qi2®. Images were analysed on Cell profiler software.

BrdU ChIP-qPCR

The same protocol as for replication timing was performed until BrdU immunoprecipitation (IP). For the BrdU-IP, 140μL of IP buffer (Tris pH8 50mM, EDTA 2mM, NaCl 300mM, Triton 1%, H2O qsp, 14mM NaOH extemporaneously) and 10μg of the monoclonal anti-BrdU antibody (BD Biosciences, 347580) were added to DNA and incubated on rotating wheel overnight at 4 °C. 1.5mg of magnetic beads (Dynabeads™ Protein G, Thermofisher 1004D) previously washed with PBS (15min on wheel at RT) and IP buffer was added to the mix and incubated on wheel 2 hours at 4°C. After washing twice with 800μL of Buffer B (Tris pH8 20mM, EDTA 2mM, NaCl 250mM, Triton 0.2%, H2O qsp) and with 800μL of Tris pH8 10mM, beads were resuspended in 100μL of Tris pH8 10mM. Immuno-precipitated DNA was then recovered by a reversion step with a solution containing 1% SDS and 0.5mg Proteinase K for 2h at 37 °C while shaking. The supernatant was incubated overnight at 65 °C while shaking. A final phenol-chloroform purification was performed and DNA concentration was measured with Nanodrop technology before performing qPCR. qPCR was performed for the specific amplification of 2 early and 2 late replicating control regions, 3 ADV aRTIL and amplicon from neo-synthesized mitochondrial DNA for normalization (Table S3). StepOne technology was used to do the qPCR. For each genomic region amplified, we quantified the percentage of S1 and S2 after normalization with mitochondrial DNA (Blin, M.; Le Tallec, B.; Nähse, V.; Schmidt, M.; Brossas, C.; Millot, G.A.; Prioleau, M.-N.; Debatisse, M. Transcription-Dependent Regulation of Replication Dynamics Modulates Genome Stability. Nature Structural & Molecular Biology 2019, 26, 58–66, doi:10.1038/s41594-018-0170-1.)
